# A Burnt-Out Health: Stigma towards Mental Health Problems as a Predictor of Burnout in a Sample of Community Social Healthcare Professionals

**DOI:** 10.3390/bs14090812

**Published:** 2024-09-13

**Authors:** Sara Zamorano, Clara González-Sanguino, Eduardo Fernández-Jiménez, Manuel Muñoz

**Affiliations:** 1 Department of Personality, Assessment and Clinical Psychology, School of Psychology, Complutense University of Madrid, 28223 Madrid, Spain; sarazamo@ucm.es (S.Z.); mmunozlo@ucm.es (M.M.); 2 Chair Against Stigma Grupo 5, Complutense University of Madrid, 28223 Madrid, Spain; 3 Department of Psychology, School of Education and Social Work, University of Valladolid, 47011 Valladolid, Spain; 4 Department of Psychiatry, Clinical Psychology and Mental Health, La Paz University Hospital, 28046 Madrid, Spain; e.fernandez.jimenez@facultyue.es; 5 Hospital La Paz Institute for Health Research (IdiPAZ), 28046 Madrid, Spain; 6 Faculty of Social Sciences and Communication, Universidad Europea de Madrid, 28670 Madrid, Spain

**Keywords:** burnout, stigma, professional skills, social healthcare professionals, working conditions, healthcare cost reduction

## Abstract

Burnout is a primary psychosocial risk factor in the workplace. Mental health stigma, which includes negative cognitions, emotions, and behaviors, also undermines the performance of social healthcare professionals. This study aimed to explore the levels of burnout in a sample of community social healthcare workers as well as its relationships with variables such as stigma towards mental health problems, professional skills, and job characteristics. An online assessment was conducted with 184 social healthcare professionals (75.5% female, mean age = 40.82 years, SD = 9.9). Medium levels of burnout and stigma and high levels of professional skills were observed. Multiple linear regression analyses revealed that stigma towards mental health problems and professional skills predicted emotional exhaustion (R^2^ = 0.153, F(4, 179) = 9.245, *p* < 0.001), depersonalization (R^2^ = 0.213, F(3, 180) = 17.540, *p* < 0.001), and personal accomplishment (R^2^ = 0.289, F(5, 178) = 15.87, *p* < 0.001). These findings suggest that social healthcare systems could benefit from taking care of the mental health of their workers by addressing burnout, tackling negative attitudes towards mental health problems, and providing professional skills training. This would help to make social healthcare systems more inclusive and of higher quality, thereby reducing health costs.

## 1. Introduction

Burnout is defined as an individual’s response to work stress, which develops progressively and can become chronic, causing alterations in mental and physical health [1,2]. It is composed of three interconnected dimensions characterized by (a) emotional exhaustion (feelings of overload and fatigue, lack of emotional and physical resources), (b) depersonalization (detachment and indifference towards work and clients), and (c) lack of personal accomplishment (negative professional self-evaluation and doubts about job skills). Burnout appears when the job demands, and unfavorable working conditions exceed the abilities of the personnel. In the healthcare context, when the workload is excessive, work shifts are irregular and long, and professional–client ratios are low, the probability of developing burnout increases [2]. In this unfavorable context, there are individual variables that moderate this relationship and interact with these stressors, which have been widely studied in social healthcare personnel [3,4]. Specifically, poor interpersonal skills in dealing with clients, many years of working under these conditions, or having low levels of self-efficacy or confidence in one’s own work influence the development of burnout.

The World Health Organization [5] has included burnout as a syndrome in the 11th edition of the International Classification of Diseases (ICD-11) and has identified it as one of the main psychosocial risks in the workplace. Globally, between 11% and 51% of healthcare workers show severe signs of burnout [4,6,7,8,9], and less than one in five reports of satisfaction with their work/life balance [10]. In Spain, a recent meta-analysis [8] estimates the overall prevalence of burnout in health professionals at 24%. Specifically, the prevalence of emotional exhaustion is 37%, depersonalization 36%, and low personal accomplishment 33%. Despite the cultural and socioeconomic differences in different countries, which may determine the structural framework of healthcare workers’ work and affect the variation in these percentages, even the lower estimates are worrying. In addition, the lack of disclosure that may occur on many occasions must also be taken into account, since recognizing a problem of this type is always difficult for a professional (social desirability, fear of repercussions, feelings of shame, etc.).

Social healthcare workers are exposed to greater physical and psychological risks than other professions, with burnout levels twice those of the general population [11,12]. This syndrome has repercussions for healthcare workers on a personal level, as they are more prone to developing symptoms of anxiety and depression, life dissatisfaction, low self-esteem, and physical problems [13]. In addition, burnout influences work performance by reducing the ability of staff to make decisions and deal with problems, with a consequent impact on productivity and the treatment of people in care [3,13]. Therefore, burnout generates a high economic cost for health and social services, estimated at a global level of USD 322 billion [14]. 

Another variable that can negatively affect the work of social healthcare personnel is stigma [15]. It is defined as a complex, multilevel social process by which one group in a position of power stereotypes and labels (attitudes, beliefs), prejudges (attributions, emotions), and discriminates (behaviors) against another group [16,17]. When stigma towards mental health problems (MHPs) occurs in a social healthcare setting, its consequences are severe. In this context, professionals may have doubts about the ability of people with MHPs to adhere to treatment or recover from physical pathologies [18,19]. Consequently, the quality of care decreases, with deficiencies in the care of medical pathologies, fewer referrals to specialists, exclusion of therapeutic decisions, coercive treatments, and erroneous attribution of physical symptoms to previous mental health problems, eclipsing physical diagnoses [18,19,20]. Social healthcare workers also experience stigma by association, as they are the target of negative stereotypes and prejudices, including doubts about their mental health and beliefs that their work is uncomfortable and dangerous [21]. In reference to the latter, stigma can also affect social healthcare workers in the first person if they have MHP.

Thus, burnout is an important variable for understanding the stigmatization of people with MHP by healthcare personnel [19]. Its components (high emotional exhaustion, depersonalization, and low personal accomplishment) are associated with negative attitudes and distant treatment of clients [2,19]. However, research on both constructs in social healthcare settings is limited. O’Connor et al. [22] found that emotional exhaustion and depersonalization are the strongest and most consistent correlates of stigmatizing attitudes among psychology doctoral students. Additionally, in studies among healthcare professionals, higher levels of depersonalization and emotional exhaustion [23,24] and less personal accomplishment [25] were associated with greater stigma. These findings have been replicated in a program for medical residents that reduced burnout and stigmatization of MHPs through role playing [26]. 

Previous studies have also established an inverse relationship, suggesting that social healthcare workers’ stigma towards people with MHPs affects the development of burnout, with greater depersonalization and emotional exhaustion [27,28,29,30,31]. In addition, among medical students, higher levels of stigma are associated with higher burnout [32]. Despite this, the influence of stigma towards mental health problems on burnout among community social healthcare professionals remains under researched. Therefore, it is essential to identify the possible key variables within this relationship to provide specific and concrete recommendations to reduce burnout among professionals in this context. This would allow robust, evidence-based interventions to be constructed and tested for effectiveness.

In view of the above, the relationship between stigma and burnout is particularly relevant. This study aimed to offer an exploratory approach to the levels of burnout among social healthcare workers and their relationship with theoretically relevant variables such as job characteristics, stigma towards MHPs, and professional competencies.

## 2. Materials and Methods

### 2.1. Procedure

This study followed a cross-sectional correlational design and was developed with the collaboration of the Cátedra UCM-Grupo 5 Contra el Estigma and its collaborating social healthcare centers. Professionals were invited via email to complete a 15 min online assessment battery. Data were collected between November 2022 and February 2023, and professionals were sent four reminders to participate. Before completing the evaluation, the participants were provided with information about the study, and their informed consent was obtained. Participation was voluntary and without compensation, and the data were coded to ensure confidentiality.

This study was approved by the Research Ethics Committee of Complutense University of Madrid (Ref: CE_20220317-07_SAL) and the Deontological Committee of Grupo 5 (Ref: FPR-PE02-07.02). The data obtained were processed in accordance with Regulation (EU) 2016/679 of the European Parliament and Council of 27 April 2016, on the protection of personal data.

### 2.2. Sample

Incidental sampling by convenience was carried out in an initial population of 3335 professionals from 159 centers of the Spanish social healthcare network, of which 195 agreed to participate in the study. The inclusion criteria were as follows: (1) active social healthcare professionals, (2) over 18 years of age, (3) with working experience, and (4) in contact with clients. After excluding participants who were inactive at the time of completion of the evaluation, the final sample size was 184.

The total sample included 184 social healthcare professionals (75.5% female; mean age = 40.82 years, SD = 9.9, range: 21–63 years). Professionals reported having worked an average of 12.34 years (SD = 8.43; range: 0–37 years) in the social healthcare field and working an average of 35.34 h per week (SD = 10.35; range: 5–80 h). In terms of mental health, 50% considered that they had first-person experience with MHPs, 97.3% knew someone with MHPs, and 49.5% lived or had lived with a person under these conditions. On the other hand, 53.8% acknowledged having had a fair amount of mental health training during their academic career, and 51.6% had received specific training on stigma and mental health in the last 12 months. The results are presented in Table 1. The results of the correlations can be found in the Supplementary Materials.

### 2.3. Variables and Instruments

#### 2.3.1. Sociodemographics

The following variables were collected: age, gender (male, female, other), employment status (active, unemployed, retired, other), profession, years of practice/experience, and average hours worked per week.

#### 2.3.2. Mental Health Information

Several dichotomous questions created ad hoc were asked with relation to the mental health of the professionals (do you consider that you have or have had a mental health problem at some time in your life?); to their contact with people with mental health problems (do you know any person in your environment who has or has had a mental health problem? do you currently live with, or have you ever lived with, a person with a mental health problem?); to their training in mental health (throughout your training, have you had subjects related to mental health?); and to mental health stigma (in the last 12 months, have you received any type of training/activity on stigma towards people with mental health problems?).

#### 2.3.3. Burnout

The Granada Burnout Questionnaire [33] (GBQ), designed to assess burnout in healthcare personnel, was used to evaluate burnout levels. This scale was specifically designed and validated for the Spanish context and is in line with the most up-to-date theoretical framework on burnout and its dimensions. This 26-item Likert-scale (1–5) questionnaire consists of three dimensions: emotional exhaustion (EE), indicating lack of energy and depletion of emotional resources and ranging from 9 to 45 points; depersonalization (DP), indicating development of indifferent and cynical attitudes towards clients, colleagues, and the organization of employment, and ranging from 7 to 35 points; and personal accomplishment (PA), indicating the evaluation by the worker of their work capacity and interest in their work and ranging from 10 to 50 points. The dimensions were calculated following the instructions of the original study, with all items corrected such that a high score signifies a high value in the evaluated trait. Therefore, higher burnout levels mean higher scores in EE and DP, but lower scores in PA. The authors proposed norms for nursing personnel, in which the 50th percentile corresponded to EE = 23, DP = 12, and PA = 42. The reliability of the three subscales in this study’s sample was EE: α = 0.861, ω = 0.862; DP: α = 0.899, ω = 0.894; and PA: α = 0.768, ω = 0.746. 

#### 2.3.4. Stigma

Healthcare Professionals’ Stigma Towards Mental Health Problems. To assess stigma towards MHPs specifically in the healthcare setting, we used the Opening Minds Stigma Scale for Health Care Providers (OMS-HC) [34] in its validated Spanish version [35]. This 20-item Likert-scale (1–5) questionnaire is one of the most widely used measures of the attitudes and intentions of healthcare personnel towards people with MHPs worldwide. Following the instructions of the authors of the original version, 15 of the 20 items were used, ranging from 15 to 75 points, with higher scores indicating greater stigma. The reliability of the scale in this sample was α = 0.810 and ω = 0.805.

Attitudes Towards Mental Health Problems. The attitudes of professionals towards people with MHP were assessed using the 12-item version of the Community Attitudes to Mental Illness (CAMI) [36], validated in Spanish [37]. This Likert-scale (1–5) questionnaire assesses the extent to which people with MHPs are tolerated, excluded, or alienated from society. It is one of the most widely used instruments to understand how society behaves towards people with MHPs. The total score was calculated by summing each item and ranges from 12 to 60 points, with higher scores indicating greater stigma. The norms set that the 50th percentile corresponded to 27 points. In this sample, the reliability of the scale was α = 0.742 and ω = 0.727.

Attributions towards Mental Health Problems. Attributions towards MHPs were measured using the Attribution Questionnaire-9 (AQ-9) [38] in its validated Spanish version [39]. This Likert-scale (1–9) questionnaire is the most relevant and used measure of negative emotions toward people with MHPs. The total score was calculated by summing each item and ranges from 9 to 81 points, with higher scores indicating higher stigma levels. In this sample, the reliability was α = 0.682 and ω = 0.895. 

Social Distance. The Social Distance Scale (SDS) [40], in its validated version in Spanish [41], was used to assess willingness to maintain social contact with people with MHPs with varying degrees of closeness (neighbor, friend, partner). In reference to stigma, the behavioral dimension is particularly important, because of the very harmful consequences it has on the life of the person discriminated against. This Likert-scale (1–5) questionnaire is composed of two dimensions: closeness and social interaction (CSI), indicating a willingness to accept a relationship with a person with MHPs and which ranges from 3 to 15 points, and intimacy and trust (IT), which indicates the degree of intimacy that one is willing to have with someone with an MHP and ranges from 2 to 10. Both dimensions indicate that the higher the score, the lower the social distance. In this sample, the reliability values for each subscale were as follows: CSI: α = 0.730, ω = 0.731 and IT: α = 0.773.

#### 2.3.5. Professional Skills

Self-efficacy. The General Self-Efficacy Scale (GSES) [42], in its Spanish validated version [43], was used to assess feelings of personal competence. This 10-item Likert-scale (1–10) questionnaire assesses the feeling of competence in coping with stressful situations. It has a wide range of applications, and is a suitable indicator of quality of life, work satisfaction, and burnout. The total score was calculated by adding the scores of the 10 items and ranges from 10 to 100 points. Higher scores indicate a greater feeling of self-efficacy. The norms indicate that the 50th percentile corresponds to 70 points. In this sample, the reliability values were α = 0.899 and ω = 0.897.

Communication Skills. The Scale on Communication Skills in Health Care Professionals (SCS-HCP) [44] was used to assess professional communication skills. The SCS-HCP is a valid and reliable instrument adjusted to the Spanish healthcare context. This 18-item Likert-scale (1–6) questionnaire is composed of the following four dimensions: informative communication (IC), indicating an ability to successfully exchange information in the attentional relationship with clients, ranging from 6 to 36 points; empathy (EM), the ability to understand the emotional states of clients, active listening, and empathic response, ranging from 5 to 30 points; respect (RE), the respect in the relationship with clients, ranging from 3 to 18 points; and assertiveness (AS), the ability to express feelings, thoughts, and needs in a skillful way in the relationship with clients, ranging from 4 to 24 points. Regarding the interpretation of the four dimensions, the higher the score, the greater the skill in each domain. The norms, designed for nursing personnel, indicate that the 50th percentile corresponds to IC = 31, EM = 24, RE = 16, and AS = 17. In this sample, the reliability values for each subscale were IC: α = 0.764, ω = 0.772, EM: α = 0.845, ω = 0.848, RE α = 0.780, ω = 0.812; and AS α = 0.546, ω = 0.551. 

### 2.4. Data Analyses

First, descriptive analyses of sociodemographic, burnout, and mental health variables were conducted. Bivariate correlations were estimated using Pearson’s test between the three burnout dimensions (dependent variables) and the continuous independent variables, as well as point-biserial correlations for categorical independent variables. Significant variables were entered into three regression models following a forward method and validated using a backward method, one for each criterion variable (EE: emotional exhaustion; DP: depersonalization; and PA: personal accomplishment). Coefficients of determination (R^2^) were computed for each regression model as an effect size index, based on Cohen’s interpretation guidelines [45], as follows: between 2–13%, small effects; between 13–26%, medium effects; and greater than 26%, large effects. Outliers were studied and not removed because they did not meet the Cook and Leverage elimination criteria [46]. SPSS software (version 29.0.2.0) was used to perform all analyses, and the significance level was set at *p* < 0.05. 

## 3. Results

### 3.1. Burnout, Stigma and Professional Abilities

The overall burnout scores revealed average levels in its three dimensions: EE, DP, and PA (EE: M = 21.79, SD = 6.98, range: 9–45; DP: M = 11.61, SD = 3.04, range: 7–35; PA: M = 40.90, SD = 5.66, range: 10–50). According to GBQ norms, 35.3% presented moderate-to-high levels of emotional exhaustion, 40.8% presented moderate-to-high levels of depersonalization, and 54.3% presented low-to-moderate levels of personal accomplishment. On the other hand, the sample scores on the OMS-HC revealed medium levels of stigma towards MHPs in the social healthcare setting (M = 38.96, SD = 8.20, range: 15–75). According to CAMI norms, 26.1% of professionals presented moderate-to-high levels of negative attitudes towards people with MHPs, with a total sample mean of 19.91 (SD = 4.94, range: 12–60). Regarding negative attributions measured using the AQ-9, low levels of negative emotionality towards people with MHPs were found (M = 26.31, SD = 7.36, range: 9–81). On the other hand, the scores on the SDS dimensions revealed high levels of disposition to closeness and social interaction, as well as moderate levels of disposition to intimacy and trust (M = 12.74, SD = 1.88, range: 3–15; M = 5.65, SD = 1.88, range: 2–10). 

Regarding the overall self-efficacy of the sample measured using the GSES, the professionals reported moderate-to-high levels of self-efficacy (M = 72.67, SD = 11.86, range: 10–100). According to the norms, 89.1% of the sample showed moderate-to-high levels of self-efficacy in coping with stressful situations. Finally, the communication skills of the professionals in the sample, as measured using the SCS-HCP, were reported to be moderate-to-high in its four dimensions: IC, EM, RE, and AS (IC: M = 31.04, SD = 3.70, range: 6–36; EM: M = 27.01, SD = 2.94, range: 5–30; RE: M = 16.4, SD = 1.60, range: 3–18; AS: M = 18.54, SD = 2.83, range: 4–24). According to the SCS-HCP norms, 73.9% of the sample reported moderate-to-high levels of informative communication, 95.6% reported moderate-to-high levels of empathy, 88.6% reported moderate-to-high levels of respect for clients, and 87.5% reported moderate-to-high levels of assertiveness. The detailed scores for this sample are presented in Table 2.

### 3.2. Predictors of Burnout among Community Social Healthcare Professionals

#### 3.2.1. Emotional Exhaustion

In all three models, the assumptions for multiple linear regression were met. The regression model on emotional exhaustion (EE) included four predictors in its final step (Table 3), achieving an R^2^ = 0.153 (moderate effect size), indicating that 15.3% of the total variance of emotional exhaustion could be explained by the model. Higher professional stigma scores toward MHPs (7.3%), lower levels of assertiveness (2.9%), and higher mean hours worked per week (1.4%) predicted greater emotional exhaustion. Not having suffered an MHP was negatively correlated with EE (3.7%).

#### 3.2.2. Depersonalization

The final regression model on depersonalization (DP) included three predictors and achieved a moderate effect size, explaining 21.3% (R^2^ = 0.213) of the total variance (Table 4). Specifically, lower levels of empathy among professionals were associated with higher DP levels (15.7%). Conversely, negative community attitudes toward MHPs (2.9%) and a higher number of years in the profession (2.7%) predicted greater depersonalization.

#### 3.2.3. Personal Accomplishment

To predict personal accomplishment (PA), the final regression model included five predictors, which accounted for 28.9% (R^2^ = 0.289) of the overall variance, achieving a large effect size (Table 5). Higher levels of empathy (18%), assertiveness (3.5%), and general self-efficacy (1.5%) predicted higher PA levels. Not having experienced a mental health problem also predicted higher PA (3.4%), whereas negative community attitudes toward MHPs predicted lower PA levels (2.5%).

## 4. Discussion

### 4.1. Theoretical Contributions

The present research aimed to examine the relationship between stigma toward people living with mental health problems (MHPs), communication skills, and other job-related variables with the different burnout dimensions among Spanish social healthcare workers.

Only around half of the sample had received specific mental health training during their careers, and only half had been trained on stigma. This is consistent with the results found on stigma measures in this sample, with average levels observed on the OMS-HC scale, and more than 25% of the sample presenting negative attitudes towards mental health problems (CAMI). Other studies have shown the presence of negative attitudes towards mental health problems among social healthcare professionals, with similar levels obtained in the OMS-HC in Mexico [47] and Germany [48]. In addition, regarding community attitudes towards MHPs, the values are comparable to other studies carried out with healthcare personnel in Spain and other countries [49]. However, in this study, the mean score on the CAMI questionnaire (M = 19.91, SD = 4.94) was significantly lower than that of the general Spanish population (M = 27.54) [50]. This agrees with other studies [19,51,52], indicating that social healthcare workers show less stigma than the general population. However, the existence of stigma toward MHPs in this population is particularly important. One of the most serious consequences of stigma at this level is the delay among users and patients seeking help, up to eight years for people with MHPs, constituting a risk factor that increases morbidity and mortality [53].

Half of the professionals in this sample had first-person experience of mental health problems, which is consistent with the moderate-to-high levels of burnout found in this study. However, the professionals’ overall perceived self-efficacy and communication skills were adequate. The results for emotional exhaustion were slightly lower than those found in a systematic review of mental health professionals by O’Connor et al. [4], where EE was estimated at 40% (vs. 35.3% in the present study). However, the levels in this sample were higher in terms of DP (22% vs. 40.8% in this study) and low PA (19% vs. 54.3% in the present study). In the national context, the results are similar to those found by Pujol-de Castro et al. [8] in a meta-analysis of burnout prevalence among 16,076 medical professionals in Spain, where EE affected almost 37% of the sample and DP levels were 36%. However, the levels of low PA were again higher in our sample (33% vs. 54.3% in our study). 

Different variables were identified as predictors of burnout among social healthcare professionals, highlighting the differences between the various dimensions of the construct. For example, in relation to EE, stigma, lower assertiveness, and more hours worked stood out as predictors. For DP, the main predictors were low empathy, worse community attitudes towards MHPs, and more years of work. For PA, the significant predictors were empathy, assertiveness, self-efficacy, not having MHPs, and community attitudes towards MHPs. 

These results are consistent with other research indicating that stigma generates greater EE and DP [27,30,31], although the present study specifies that stigmatizing attitudes and behavioral intentions specifically affect EE and that community attitudes towards MHPs have a greater relationship with DP and, to a lesser extent, with PA. This indicates the importance of attitudes and emotions in the emotional dimension of burnout. It also points to the benefits of work environments in which mental health is understood not only from a biological point of view but also from a community point of view, where the development of cynical and indifferent attitudes is avoided, which would otherwise increase DP levels. In this line, mental health training for all social healthcare workers could help them maintain a realistic idea of what MHPs are and how they can affect the people they care for and themselves, thus increasing their knowledge and confidence in working with this population. Several studies have emphasized that communication and clinical skills training for professionals can reduce their stigmatizing attitudes by increasing their sense of self-efficacy and their knowledge of MHPs. In addition, direct and positive contact with people with lived experiences in mental health can improve attitudes toward them [19], and face-to-face and contact-based interventions are particularly effective at reducing stigma [54].

Other important predictors are professional skills, such as assertiveness, empathy, and general self-efficacy. In this study, these variables are postulated to protect against burnout. Specifically, assertive communicative attitudes are negatively related to emotional exhaustion. Empathy is a professional skill that appears to be a protective variable against depersonalization. Finally, assertive and empathic attitudes, together with higher perceived general self-efficacy, allow professionals to develop greater personal accomplishment. Thus, training in this type of professional skills is postulated as a key intervention target in the prevention and management of burnout syndrome among social healthcare workers. These results are in line with previous studies that point to training in professional skills, such as empathy and assertiveness, as protective against both stigma and burnout [24,31].

Job-related factors, such as hours worked and years of experience, also emerged as predictor variables. In this case, more hours worked and more experience predicted higher levels of emotional exhaustion and depersonalization, respectively. This is consistent with previous studies that found higher levels of burnout among professionals with long careers and greater job demands, such as time pressure and long shifts [31,55]. However, these variables seem to have less weight in the development of burnout than stigma towards MHPs or professional skills.

Finally, the experience of mental health problems throughout life also influences how burnout develops in this population. In particular, professionals who have had an MHP showed higher emotional exhaustion levels, while the lack of any psychological problems could be a protective factor that would allow them to achieve more personal accomplishment. These findings agree with previous studies, which shows that professionals with depression and anxiety have higher degrees of burnout [56]. 

### 4.2. Practical Implications and Recommendations

In general, the strategies proposed so far in Spain to address burnout in social healthcare settings are related to changes in working conditions (breaks, work–life balance, fixed schedules, diversification of tasks), development of tools for psychological wellbeing, expansion of social support, and extension of leisure time [8]. It is clear that the improvement of structural working conditions, such as adequate shifts and acceptable user/professional ratios, is fundamental, but the identification of predictor variables of burnout that seem to be key in this population is important for prioritizing both primary and secondary preventive interventions. These interventions could be aimed at reducing negative attitudes towards MHPs and providing professional skills training to generate more knowledge and confidence among these workers.

Considering these predictors of each of the burnout dimensions, specific approaches could be developed. Emotional exhaustion, depersonalization, and the low personal accomplishment of professionals are related to their levels of stigma, their perception of self-efficacy, and their professional skills, but also to their working conditions, the length of time they have been working, and their own mental health status.

Stigma is a variable that not only affects the care that professionals must provide, but also has implications for their own wellbeing. Stigma-free workplaces for users and workers will create a work climate less conducive to the onset of burnout. 

This requires policies that structurally protect workers and encourage the acceptance and promotion of mental health. Programs and campaigns to raise awareness about stigma and, in general, to promote the importance of good mental health can help create healthier working environments, where disclosure of MHPs is not taboo and the necessary help is obtained as early as possible, along with good peer support. 

Additionally, education courses on stigma for professionals, and equipping those professionals with more communication skills to use in the daily care of their users, can be combined with the traditional wellbeing strategies mentioned above. These courses could be implemented at the basic level of university education or from the companies themselves as part of the necessary professional training.

In short, it is important to design intervention programs that combine empirically validated stigma reduction strategies (such as mental health literacy and contact with people with MHPs) and training in communication skills with an emphasis on assertiveness and empathy. In addition, these programs would benefit from including self-care strategies promoted not only by professionals but also by the structure of the organization in which they work, which should provide these professionals with adequate working conditions.

### 4.3. Limitations of the Study and Further Research

The present study had several limitations. First, it has a cross-sectional design, which prevents us from establishing temporal sequences between variables and drawing solid causal associations. In addition, incidental sampling may have limitations in terms of generalizing the results of the study to the population of social healthcare personnel, which is difficult to access when conducting research, in turn justifying the limited sample size of this study. The sample was heterogeneous and all the participants worked in a community social healthcare setting, which also restricts the generalizability of our results. Additionally, the norms of the burnout and professional skills scales were built from samples of nurses, which limits the generalizability of the results to the population of the present study.

Longitudinal studies are required in the future to understand the processes of change in burnout in social healthcare settings. Additionally, studies with wider and more representative samples are needed to obtain results that can be better generalized to the population. In addition, stigma measures should be specifically validated among social healthcare professionals in further studies in order to obtain results adjusted to the differential characteristics of this population.

Finally, it will be interesting to understand the effects on the different burnout dimensions of anti-stigma programs for healthcare workers, as well as the impact of policies and structural modifications that imply the creation of stigma-free centers.

## 5. Conclusions

Stigma towards MHP and professional skills, such as assertiveness or empathy, were found to be the main predictors of burnout in this study of healthcare professionals. Other variables of lesser importance are work-related characteristics, such as average hours worked per week and years of professional experience. Stigma is not only a harmful variable for users of health care facilities, but also for their professionals. One strategy to prevent and intervene in burnout among professionals is to address stigma and to work on those professionals’ attentional skills. Health and social care settings can benefit from these strategies and move towards more inclusive health and social care systems that are both person-centered and professional-centered, reducing the health care costs associated with burnout and its impact on productivity.

## Figures and Tables

**Table 1:** Sociodemographic characteristics of the sample and mental health information.

Variable (Range)	N	%
Age (21–63)		
21–30	32	17.4
31–45	88	47.8
>46	64	34.8
Gender		
Male	45	24.5
Female	139	75.5
Years of practice (0–37)		
0–5	51	27.7
6–15	71	38.6
>16	62	33.7
Hours worked per week (5–80)		
5–20 h	20	10.9
21–37.5 h	80	43.5
>38 h	84	45.7
Do you consider that you have, or have had at some point in your life, a mental health problem? (Own MHP)		
Yes	92	50
No	92	50
Do you know anyone in your environment who has or has had a mental health problem? (Knowing someone with MHP)		
Yes	179	97.3
No	5	2.7
Do you currently live with, or have you ever lived with, a person with a mental health problem? (Living with someone with MHP)		
Yes	91	49.5
No	93	50.5
Throughout your training, have you had any mental health related subjects? (MH training)		
Many	99	53.8
Few	85	46.2
In the last 12 months, have you received any training/activities on stigma towards people with mental health problems? (Stigma training last 12 months)		
Yes	95	51.6
No	89	48.4
**Total**	**184**	**100**

**Table 2:** Burnout (GBQ), stigma (OMS-HC; CAMI; AQ-9; SDS), and professional abilities (GSES; SCS-HCP) by sociodemographic characteristics and mental health information.

Variables (Range)	GBQ						SDS	
	EE (9–45)	DP (7–35)	PA (10–50)	OMS-HC	CAMI	AQ-9	CSI (3–15)	IT (2–10)
Total sample	21.79 (6.98)	11.61 (3.04)	40.90 (5.63)	38.96 (8.20)	19.91 (4.94)	26.31 (7.36)	12.74 (1.88)	5.65 (1.88)
Age								
21–30	21.03 (7.17)	10.69 (3.05)	41.53 (5.56)	37.06 (5.69)	17.91 (3.62)	24.31 (5.47)	13.13 (1.60)	5.81 (2.33)
31–45	22.44 (7.33)	11.39 (2.94)	40.52 (6.12)	39.57 (8.88)	20.57 (4.84)	27.49 (7.33)	12.65 (1.99)	5.34 (1.66)
>46	21.28 (6.40)	12.38 (3.05)	41.09 (4.99)	39.06 (8.27)	20.02 (5.43)	25.69 (7.99)	12.67 (1.85)	6.00 (1.87)
Gender								
Male	22.91 (7.31)	12.49 (2.94)	39.78 (6.57)	40.93 (8.98)	21.49 (4.68)	27.36 (7.41)	12.38 (1.75)	5.82 (1.75)
Female	21.43 (6.86)	11.32 (3.03)	41.26 (5.27)	38.31 (7.87)	19.40 (4.93)	25.97 (7.33)	12.86 (1.91)	5.60 (1.92)
Years of practice (0–37)								
0–5	20.41 (5.92)	10.61 (2.94)	41.75 (4.98)	37.59 (7.10)	19.27 (4.82)	25.04 (6.64)	13.33 (1.58)	6.14 (1.94)
6–15	22.83 (7.03)	11.79 (2.83)	40.17 (6.12)	39.65 (8.34)	20.30 (5.02)	26.59 (7.57)	12.38 (2.04)	5.11 (1.58)
>16	21.74 (7.62)	12.23 (3.21)	41.03 (5.54)	39.29 (8.86)	20.00 (4.98)	27.03 (7.64)	12.66 (1.83)	5.87 (2.01)
Hours worked per week (5–80)								
5–20 h	19.00 (6.62)	11.00 (2.69)	42.10 (5.37)	36.10 (7.56)	17.90 (4.54)	25.04 (6.64)	12.95 (1.82)	6.00 (2.38)
21–37.5 h	21.21 (6.74)	11.58 (3.16)	40.45 (5.39)	37.74 (8.18)	19.51 (4.82)	26.59 (7.57)	12.86 (1.82)	5.70 (1.80)
>38 h	23.01 (7.11)	11.79 (3.02)	41.04 (5.93)	40.80 (8.05)	20.77 (5.01)	27.03 (7.64)	12.57 (1.96)	5.52 (1.83)
Own MPH								
Yes	23.11 (7.55)	11.78 (3.31)	39.96 (6.02)	38.55 (7.76)	19.50 (4.73)	26.83 (7.70)	12.77 (1.72)	5.62 (1.83)
No	20.48 (6.13)	11.43 (2.75)	41.84 (5.08)	39.36 (8.65)	20.33 (5.13)	25.79 (7.00)	12.71 (2.04)	5.68 (1.94)
Knowing someone with MHP								
Yes	21.80 (7.05)	11.54 (2.98)	41.01 (5.62)	38.74 (8.11)	19.72 (4.73)	26.07 (7.19)	12.78 (1.86)	5.66 (1.90)
No	21.40 (3.97)	14.20 (4.55)	36.80 (5.07)	46.60 (8.85)	26.80 (7.73)	35.00 (8.97)	11.40 (2.30)	5.40 (0.55)
Living with someone with MHP								
Yes	21.93 (6.86)	11.19 (3.35)	41.13 (5.64)	38.14 (7.90)	19.16 (4.59)	26.37 (7.06)	12.81 (1.96)	5.82 (1.99)
No	21.66 (7.13)	12.02 (2.67)	40.67 (5.65)	39.75 (8.46)	20.65 (5.18)	26.25 (7.67)	12.67 (1.81)	5.48 (1.76)
MH training								
Many	22.18 (6.87)	11.63 (3.03)	41.12 (5.49)	38.29 (8.32)	19.50 (4.91)	25.86 (6.96)	12.86 (1.94)	5.64 (1.88)
Few	21.34 (7.13)	11.59 (3.08)	40.64 (5.82)	39.73 (8.05)	20.39 (4.96)	26.84 (7.80)	12.60 (1.81)	5.67 (1.89)
Stigma training last 12 months								
Yes	21.26 (6.76)	11.42 (2.77)	41.41 (5.81)	38.09 (8.80)	19.18 (4.87)	25.29 (6.60)	12.77 (1.93)	5.94 (1.72)
No	22.36 (7.20)	11.81 (3.31)	40.35 (5.42)	39.88 (7.45)	20.70 (4.92)	27.39 (7.98)	12.71 (1.84)	5.35 (2.00)
**Variables (Range)**	**SCS-HCP**							
	**GSES (10–100)**		**IC (6–36)**		**EM (5–30)**		**RE (3–18)**	**AS (4–24)**
Total sample		72.67 (11.86)		31.04 (3.70)	27.01 (2.94)		16.41 (1.60)	18.54 (2.83)
Age								
21–30		73.78 (10.41)		31.91 (3.03)	28.03 (1.97)		16.88 (1.26)	19.06 (2.69)
31–45		74.34 (10.13)		30.85 (3.62)	26.88 (3.02)		16.28 (1.68)	18.31 (3.00)
>46		69.81 (14.15)		30.88 (4.10)	26.69 (3.16)		16.36 (1.61)	18.59 (2.64)
Gender								
Male		73.91 (10.56)		30.49 (4.16)	26.27 (3.08)		16.27 (1.63)	18.49 (2.86)
Female		72.27 (12.25)		31.22 (3.54)	27.25 (2.87)		16.46 (1.59)	18.55 (2.83)
Years of practice (0–37)								
0–5		75.49 (9.16)		30.94 (3.68)	27.04 (2.87)		16.61 (1.43)	18.53 (2.82)
6–15		72.73 (10.99)		30.85 (3.65)	27.03 (3.01)		16.23 (1.77)	18.48 (2.81)
>16		70.27 (14.21)		31.35 (3.82)	26.97 (2.98)		16.47 (1.51)	18.61 (2.89)
Hours worked per week (5–80)								
5–20 h		73.70 (15.59)		32.15 (4.23)	27.70 (2.92)		16.75 (1.33)	18.35 (2.50)
21–37.5 h		71.36 (11.88)		31.10 (3.75)	27.29 (2.88)		16.61 (1.61)	18.36 (2.85)
>38 h		73.67 (10.82)		30.73 (3.52)	26.58 (2.98)		16.14 (1.61)	18.75 (2.89)
Own MPH								
Yes		72.57 (11.72)		31.29 (3.41)	27.20 (2.71)		16.47 (1.46)	18.79 (2.75)
No		72.77 (12.05)		30.79 (3.98)	26.83 (3.16)		16.36 (1.73)	18.28 (2.89)
Knowing someone with MHP								
Yes		72.68 (11.87)		31.17 (3.65)	27.15 (2.83)		16.49 (1.53)	18.60 (2.81)
No		72.20 (12.54)		26.60 (3.05)	22.00 (2.55)		13.80 (1.79)	16.40 (2.70)
Living with someone with MHP								
Yes		74.03 (11.63)		31.56 (3.65)	27.53 (2.55)		16.54 (1.42)	18.97 (2.78)
No		71.33 (11.98)		30.54 (3.71)	26.51 (3.22)		16.29 (1.75)	18.12 (2.82)
MH training								
Many		71.55 (12.85)		31.32 (3.23)	27.31 (2.63)		16.42 (1.55)	18.58 (2.66)
Few		73.98 (10.50)		30.72 (4.19)	26.66 (3.25)		16.40 (1.66)	18.49 (3.02)
Stigma training last 12 months								
Yes		71.93 (13.30)		31.27 (3.68)	27.20 (2.80)		16.49 (1.50)	18.67 (2.61)
No		73.46 (10.11)		30.80 (3.74)	26.81 (3.10)		16.33 (1.70)	18.39 (3.04)

**Table 3:** Predictors of emotional exhaustion of community social healthcare professionals.

Variable	*B*	*SE*	*95% CI*	*β*	*p*
Constant	25.35	5.042	[15.40, 35.30]	-	<0.001
OMS-HC	0.17	0.06	[0.05, 0.29]	0.20	0.007
MHPs	−2.98	0.95	[−4.86, −1.11]	−0.21	0.002
AS	−0.49	0.18	[−0.84, −1.14]	−0.20	0.007
Hours worked per week	0.09	0.05	[0.00, 0.19]	0.14	0.049

Note. R^2^ = 0.153, F(4, 179) = 9.245, *p* < 0.001.

**Table 4:** Predictors of depersonalization of community social healthcare professionals.

Variable	*B*	*SE*	*95% CI*	*β*	*p*
Constant	17.34	2.54	[12.34, 22.37]	-	<0.001
EM	−0.33	0.07	[−0.48, −0.18]	−0.32	<0.001
CAMI	0.12	0.04	[0.03, 0.21]	0.20	0.007
Years of practice	0.06	0.02	[0.02, 0.11]	0.18	0.008

Note. R^2^ = 0.213, F(3, 180) = 17.540, *p* < 0.001.

**Table 5:** Predictors of personal accomplishment among social healthcare professionals.

Variable	*B*	*SE*	*95% CI*	*β*	*p*
Constant	16.45	5.00	[6.59, 26.31]	-	<0.001
EM	0.52	0.14	[0.23, 0.80]	0.27	<0.001
MHPs	2.40	0.70	[1.01, 3.79]	0.21	<0.001
AS	0.33	0.15	[0.04, 0.62]	0.16	0.026
CAMI	−0.21	0.08	[−0.36, −0.05]	−0.18	0.009
GSES	0.07	0.03	[0.01, 0.13]	0.14	0.029

Note. R^2^ = 0.289, F(5, 178) = 15.87, *p* < 0.001.

## Data Availability

The data presented in this study will be made available by the authors upon reasonable request.

## Supplementary Materials

The supporting information can be downloaded at: https://www.mdpi.com/article/10.3390/bs14090812/s1, Table S1. Bivariate correlations and point biserial correlations between the three burnout dimensions (dependent variables) and the continuous independent variables.
