# Supplementary material for: A Burnt-Out Health: Stigma towards Mental Health Problems as a Predictor of Burnout in a Sample of Community Social Healthcare Professionals
Source: Behav Sci (Basel). 2024 Sep 13;14(9):812. doi: 10.3390/bs14090812 (PMC11429299; doi:10.3390/bs14090812)
Supplement: Supplementary file 1 [file behavsci-14-00812-s001.zip › behavsci-3139101-supplementary.pdf]

Supplementary Materials

Table S1. Bivariate correlations and point biserial correlations between the three burnout dimensions (dependent variables) and the continuous independent variables.

Variable	*Emotional exhaustion*	*Depersonalization*	*Personal accomplishment*
Age	-0.008	**0.228****	-0.023
Gender	-0.091	**-0.165***	0.113
Years of practice	0.055	**0.180***	-0.027
Hours worked per week	**0.192****	0.077	-0.025
Own MPH	**-0.189***	-0.077	0.**167***
Knowing some-one with MHP	-0.009	0.143	-0.122
Living with someone with MHP	-0.020	0.137	-0.041
MH training	-0.060	-0.006	-0.043
Stigma training last 12 months	0.079	0.064	-0.094
OMS-HC	**0.280****	**0.302****	**-0.326****
CAMI	**0.205****	**0.335****	**-0.336****
AQ-9	**0.230****	**0.145***	**-0.242****
SDS-CSI	**-0.190****	**-0.217****	**0.238****
SDS-IT	**-0.135**	**-0.079**	**0.177***
GSES	-0.033	**-0.205****	**0.243****
SCS-HCP-IC	**-0.250****	**-0.297****	**0.400****
SCS-HCP-EM	**-0.230****	**-0.402****	**0.429****
SCS-HCP-RE	**-0.250****	**-0.372****	**0.354****
SCS-HCP-AS	**-0.246****	**-0.163***	**0.364****

Note. MHP = Mental health problem; MH = Mental health; OMS-HC = Opening Minds Stigma Scale for Health Care Providers (OMS-HC; Modgill et al., 2014); CAMI = Community Attitudes to Mental Illness (CAMI; Taylor & Dear, 1981); AQ-9 = Attribution Questionnaire-9 (AQ-9; Corrigan et al., 2014; SDS-CSI = Social Distance Scale (SDS; Link et al., 1987), closeness and social interaction subscale; SDS-IT = Social Distance Scale (SDS; Link et al., 1987), intimacy and trust subscale; GSES = General Self-Efficacy Scale (GSES; Baessler and Schwarcer, 1996); SCS-HCP-IC = Scale on Communication Skills in Health Care Professionals (SCS-HCP; Leal-Costa et al., 2016), informative communication subscale;); SCS-HCP-EM = Scale on Communication Skills in Health Care Professionals (SCS-HCP; Leal-Costa et al., 2016), empathy subscale;); SCS-HCP-RE = Scale on Communication Skills in Health Care Professionals (SCS-HCP; Leal-Costa et al., 2016), respect subscale;); SCS-HCP-AS = Scale on Communication Skills in Health Care Professionals (SCS-HCP; Leal-Costa et al., 2016), assertiveness subscale. Significant correlations between variables are marked in bold in the table. **. Correlation is significant at the 0.01 level (bilateral). *. Correlation is significant at the 0.05 level (bilateral).
